# A vascular image registration method based on network structure and circuit simulation

**DOI:** 10.1186/s12859-017-1649-1

**Published:** 2017-05-02

**Authors:** Li Chen, Yuxi Lian, Yi Guo, Yuanyuan Wang, Thomas S. Hatsukami, Kristi Pimentel, Niranjan Balu, Chun Yuan

**Affiliations:** 1 Department of Electronic Engineering, Fudan University, Shanghai, 200433 China; 2 Department of Electrical Engineering, University of Washington, Seattle, WA 98195 USA; 3 Department of Surgery, University of Washington, Seattle, WA 98195 USA; 4 Department of Radiology, University of Washington, Seattle, WA 98195-7115 USA

**Keywords:** Vascular image registration, Graph-based registration, Network structure, Circuit simulation

## Abstract

**Background:**

Image registration is an important research topic in the field of image processing. Applying image registration to vascular image allows multiple images to be strengthened and fused, which has practical value in disease detection, clinical assisted therapy, etc. However, it is hard to register vascular structures with high noise and large difference in an efficient and effective method.

**Results:**

Different from common image registration methods based on area or features, which were sensitive to distortion and uncertainty in vascular structure, we proposed a novel registration method based on network structure and circuit simulation. Vessel images were transformed to graph networks and segmented to branches to reduce the calculation complexity. Weighted graph networks were then converted to circuits, in which node voltages of the circuit reflecting the vessel structures were used for node registration. The experiments in the two-dimensional and three-dimensional simulation and clinical image sets showed the success of our proposed method in registration.

**Conclusions:**

The proposed vascular image registration method based on network structure and circuit simulation is stable, fault tolerant and efficient, which is a useful complement to the current mainstream image registration methods.

## Background

Image registration is an image processing step that matches two or more images of different imaging devices at multiple shooting times and angles, or from the same scene for image fusion, expression and analysis [1, 2]. Applying image registration to vascular images by fusing and reflecting same vascular structures captured from multiple images to the same display can greatly help clinical diagnosis. For example, the registration between magnetic resonance angiographic images and digital subtraction angiographic images can provide useful complementary information for physicians during treatments [3].

For the vascular image registration, due to the complicated imaging mechanism or diversified biological structures, noises and differences pose challenges to the registration. Medical images often contain complex noise which decreases the imaging quality of vascular targets, such as Gaussian noise from the sensitivity of the sensors and salt-and-pepper noise from the medical imaging equipment [4]. In addition, due to the diversity in imaging mechanism, the objects captured in different imaging modalities are not similar in appearance. Computed tomography is superior in confirming the presence of calcification [5]. However, irregular intravascular calcification plaque and blood turbulence may lead to image artifacts in Magnetic resonance Imaging (MRI) [6]. Even for the same imaging modality, such as MRI, scanning the same subject with different machines or parameters at different time can also be different [7].

To solve these challenges, many vascular image registration methods have been proposed, which can be categorized into three approaches.

For area-based registration approaches, such as cross-correlation matching [8, 9] and mutual information [10, 11] methods, the original image is directly used to estimate correspondences, which is easily influenced by noises or trapped in the local extremum. For feature-based approaches, a description of invariance (correlation coefficient [12], closed region [13], geometric features [14], coherent point drift [15]) is obtained from the image through different feature extraction methods for registration. However, due to the lack of significant features in vascular structures, it is difficult to find stable feature points in registration.

Different from previous two approaches, graph-based approach is a novel approach by extracting node properties from relatively stable geometric features and topological structures of the vascular images. Most of these methods rely on the Euclidean distance or spherical distance between feature points in the graphs network [16, 17], which are able to match vascular structures with only slight non-linearity. But when large non-linear deformation or topology missing exists, it is difficult to get a satisfactory result. A more advanced method using graph-based approach is active searching [18], which iteratively searches possible corresponding points in graph network with Gaussian process regression model and predicts more corresponding points. This method can register images with large difference, but it is complicated and computationally expensive, which is difficult to apply on large images.

In this paper, we propose a novel vascular image registration method to effectively and efficiently solve the problem of matching large scale vascular images with large non-linear difference. Our proposed method is based on graph approach, taking advantage of geometric features and topological structures of the vascular images. Our main contributions are 1) we determine structural salient points in graph network by Network Structure Index(NSI), a feasible index calculated from network structure, which avoids complicated calculation of iteratively searching possible corresponding points in active searching method. 2) A novel circuit conversion model is proposed to match the large non-linear differences in registration, in which the spatial difference in graph is reflected by voltage deviation. 3) Network decomposition based on NSI and branch sequential matching criteria is used to deal with large scale graph network. Our methods are effective and efficient in solving the problems of matching large non-linear differences in large scale graph network where previous methods based on graph-based approaches failed.

## Method

**Fig. 1:**
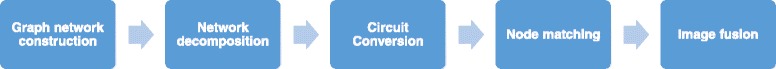
Flow chart of our method

There are five main steps in our method, as shown in Fig. 1. Firstly, both reference and sensed images are converted to graph networks, which are decomposed by Network Structure Index (NSI). Then, branches segmented from network are matched by sequential matching criteria. Afterwards, a circuit conversion model is used to further convert graph network to circuit. Finally, the voltage response is used for node matching and image fusion.

Input data which could be processed by our method can be either two-dimensional or three-dimensional vascular images. Imaging modality is not restricted as long as there is enough contrast between foreground and background. High resolution is preferred for better precision.

### Graph network construction

**Fig. 2:**
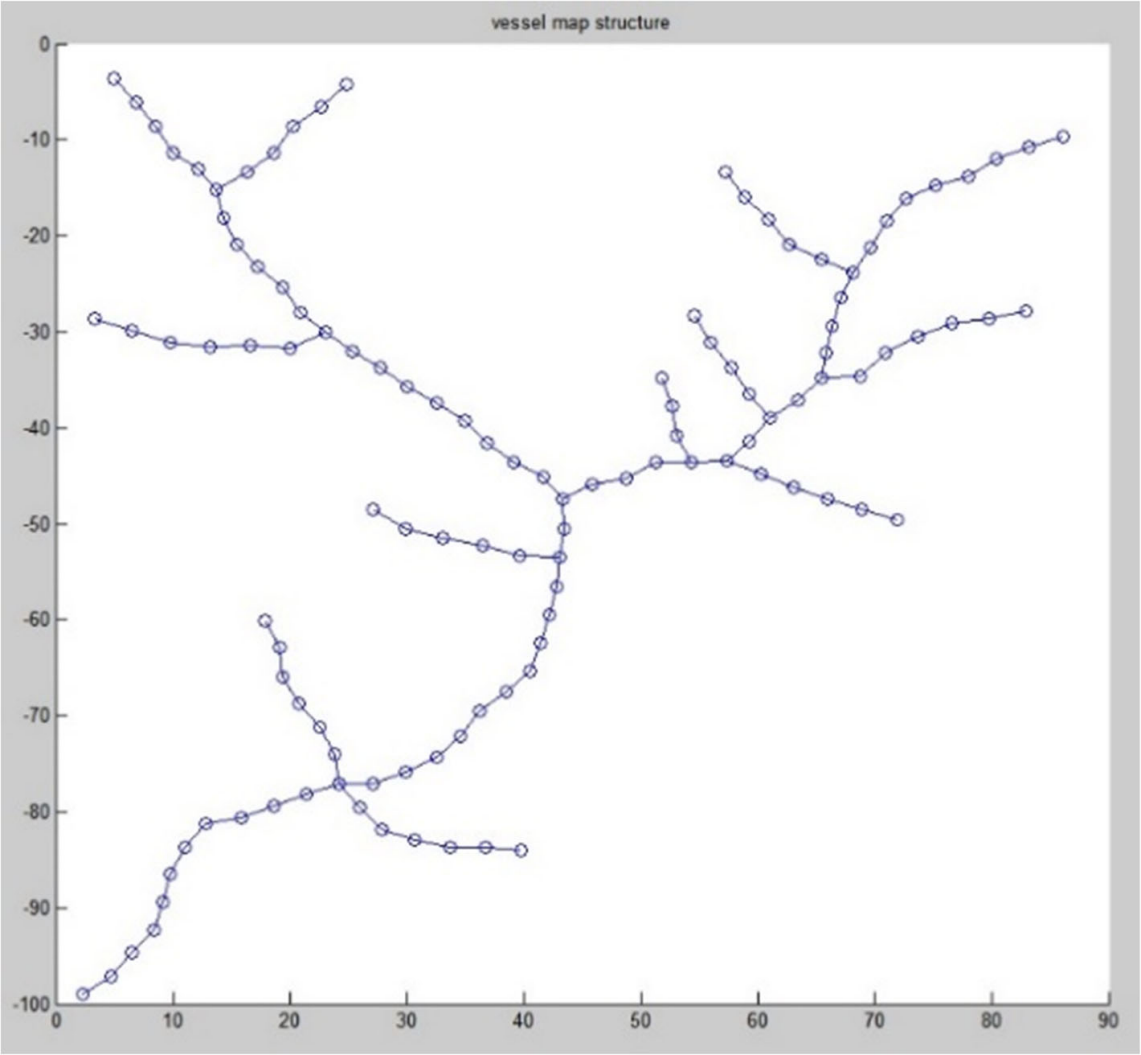
Graph network of a vascular image after vessel segmentation

Firstly, the reference image and sensed image are preprocessed by region of interest selection and image resampling for three-dimensional anisotropic images. Then, the vascular structures are traced by an open-curve snake tracing method [19, 20]. The vascular structures are used to create graph network with the radius of points in vascular structures as the node weight. A graph network is shown in Fig. 2.

### Application of network structure index (NSI)

After constructing the graph network, the network decomposition is an effective way to reduce the computational complexity. In our method, structurally salient points in the network are determined for decomposition. The structural saliency of a node should not only include the weight of the point *W*(*v*), but also need to comprise the number of edges connected to it (the degree of the node) and the value of surrounding neighbors.

The degree of node is the most commonly used structural indicator. But it is difficult to reflect the local influence and connectivity of the node and fails to determine the correspondence where a branch is missing or there is a small interference branch in the neighborhood of the branch point. The more accurate betweenness centrality [21, 22], on the other hand, is complicated in calculation and depends on the global information in network, which is sometimes unavailable when vascular image is large in dimension or some structures are missing.

In order to reflect the regional structural saliency of each point with consideration of its connection, value and influence in network, a novel Network Structure Index (NSI) has been proposed in our previous work [23]. By calculating the connectivity and weights in the neighborhood of a node, an index that is more stable and accurate than the node degree as well as more feasible than the betweenness centrality is obtained. In this paper, we use this index to detect and match structurally salient points for network decomposition.

**Fig. 3:**
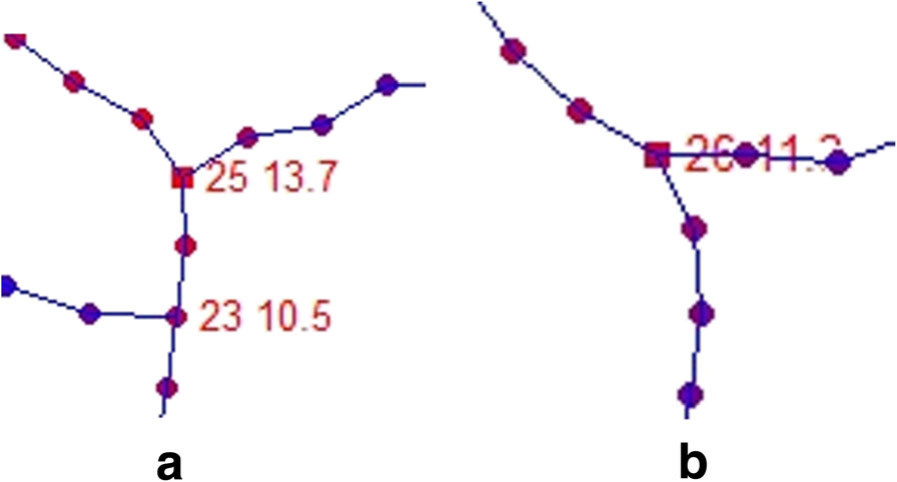
Example of a pair of corresponding structurally salient points (in square shape with id number and NSI value marked beside, color of node represent its NSI value, redder of node means higher NSI) (**a**) is from reference image, (**b**) is from sensed image

It is regulated that the structurally salient points have degree of at least three. The principle of matching structurally salient points is that the node with largest NSI in a given neighborhood (5% image size, adjustable according to vessel size and density) of reference image has a corresponding node with largest NSI in the same neighborhood of sensed image. Figure 3 shows a pair of corresponding structurally salient points, the number next to nodes is its id and NSI value. With two structurally salient nodes in neighborhood from Fig. 3a, node 23 cannot become the corresponding structurally salient point to node 26 in Fig. 3b due to its smaller NSI value compared with node 25 in Fig. 3a.

### Network decomposition

Since correspondence of structurally salient nodes has been established with NSI, the network can be decomposed into branches and the branches separated from each network need to be matched. In our method, firstly the network is decomposed by selecting connected traces between nearest corresponding structurally salient points. Secondly, traces connecting from corresponding structurally salient points on one end and node of which degree is one on the other end in shortest path are selected. Such decomposition sequence is able to avoid selecting the same branch for multiple times and to ensure that the traces selected does not contain any structurally salient points.

**Fig. 4:**
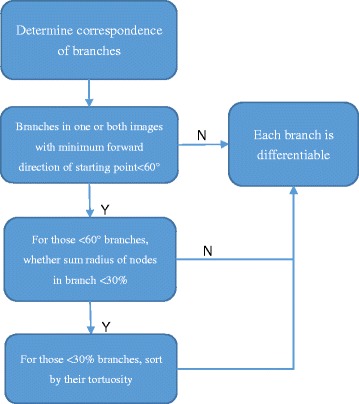
Flow chart of sequential branch matching criteria

The first part of branches in selection can be matched according to their identical corresponding structural salient points. However, to match branches from second part of selection, more information is needed. Here, the forward direction of starting point, the sum radius of nodes in branch and the tortuosity of branch are sequentially used as the criteria. Flow chart shown in Fig. 4.

The forward direction of starting point is the advancing direction from the salient point to the first node of the branch, where the counterclockwise angle from the positive *x* direction is used for measurement. In our method, 60° is used as the maximum allowable angular difference during matching. If the difference of this parameter between branches is within 60°, we should use further criteria in determining the correspondence of branches.

The sum radius of nodes can be approximated as the volume of the vessel, due to the relative slow change in radius of the nodes in branches. Here, a difference of 30% is used as the allowable amount for judgment. If that parameter is still not discriminative enough for matching branches, we should consider the last criteria.

The tortuosity of branch is the ratio of the distance accumulated between adjacent nodes in the branch to the straight distance of the branch from the beginning node to the end of node. For the vessels with large curves, their tortuosity is high. As the final criterion, we only compare the relative amount of tortuosity from several branches for judgment.

### Circuit conversion model

After the correspondence of branches in reference and sensed image is established, the nodes in each corresponding branch are matched. Due to the possible variations of vascular structures in reference and sensed images, the node matching must be flexible and robust. Conventional methods using Euclidean distance or spherical distance [16, 17] are very sensitive to the small length changes inherent to the biological structures. In vascular images, there are usually small interference branches near bifurcations, and branches may have partial missing due to low image quality, thus such methods are not suitable for more challenging registration. The idea of using circuit to simulate graph network is enlightened from circuit simulation method [24, 25] which is used to convert graph to circuit in solving graph isomorphism problems in a simple and robust approach. We found the idea of designing a circuit conversion model for registration of nodes in graph network promising, for a well-designed circuit is robust to certain damage in element, it is predictable that a well-built conversion modal is able to use the strength of circuit in registration in a same way.

**Fig. 5:**
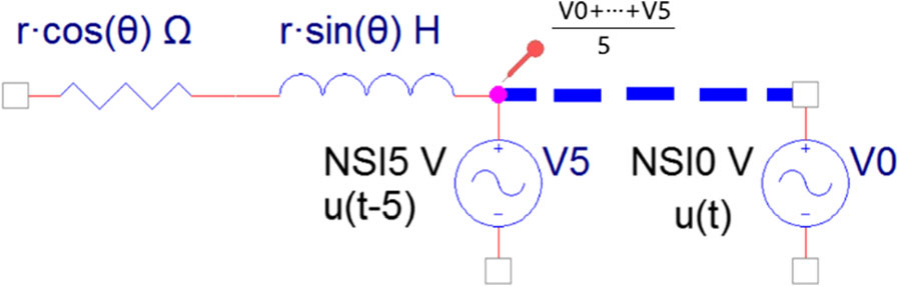
Circuit illustration of a branch by using circuit conversion model

The circuit conversion model we proposed is that each node is converted into a group of circuit elements (combination of resistance, inductance and voltage source). These elements are connected to form a circuit based on graph connection, in which the nodal response voltage is obtained by power excitation in circuit. Take the fifth node of a branch as an example, the circuit conversion result is shown in Fig. 5.

For each node in a branch, three important parameters are converted into circuit elements, the radius of the node *r*, the forward direction of the node deviated from the forward direction of the whole branch *θ*, and the NSI value of node. Considering (1) the generally irrelevance of curve and radius of vascular structures; (2) the use of complex voltage number to present voltage is available in Laplacian domain for circuit analysis; (3) the chain-like structure of the vascular node needs to use inductance to represent the imaginary part of voltage. Therefore, the nodes are converted to a combination of resistance and inductance to meet the following relationship:

1 $$ \left\{\begin{array}{l} R= r\cdot \cos \left(\theta \right)\\ {} jwl= r\cdot \sin \left(\theta \right)\end{array}\right. $$

For voltage acquisition, each voltage source is excited at time *t* = *t*_*i*_, respectively. The voltage response of the node is defined as the average nodal voltage from all the response collected before the excitation till *t*_*i*_, aiming at reducing the instabilities. Each voltage response of node in a branch constitute the integrated voltage sequence of this branch. The nodal voltage acquired for the fifth node is shown by the probe in Fig. 5.

**Fig. 6:**
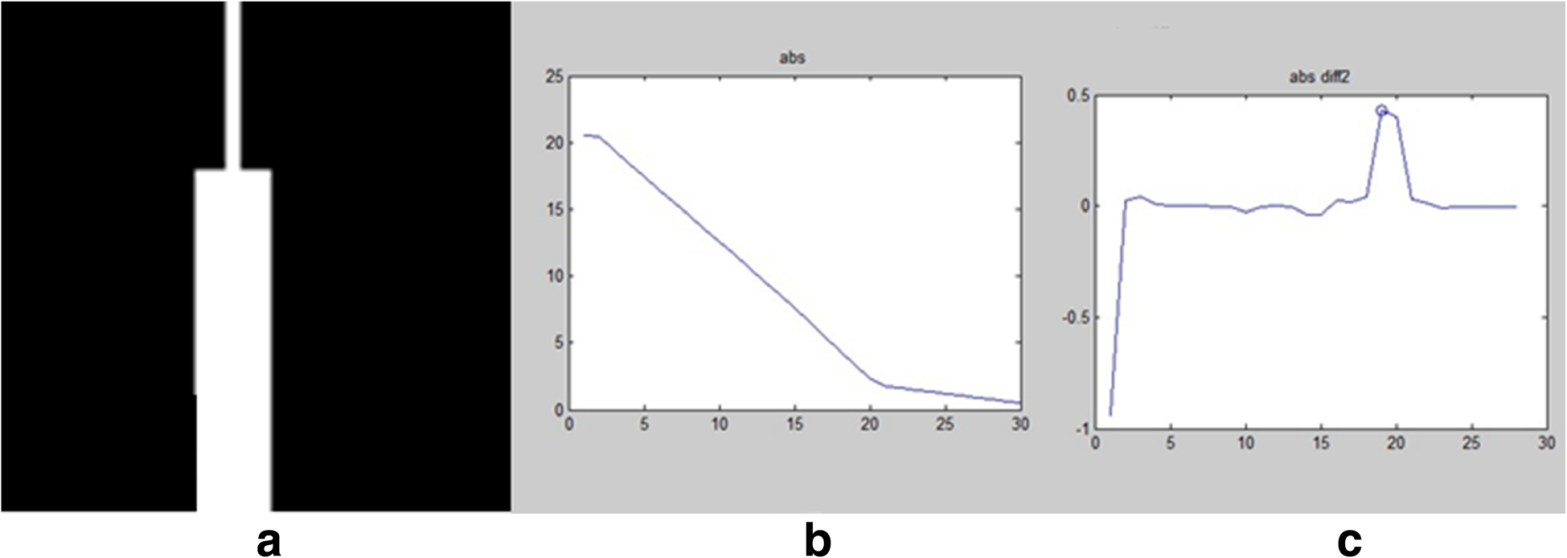
**a** A simulated vessel with thickness changed in middle. **b** The absolute value of its integrated voltage sequence. **c** The derivative absolute value of its integrated voltage sequence (thickness changing point marked in circle)

**Fig. 7:**
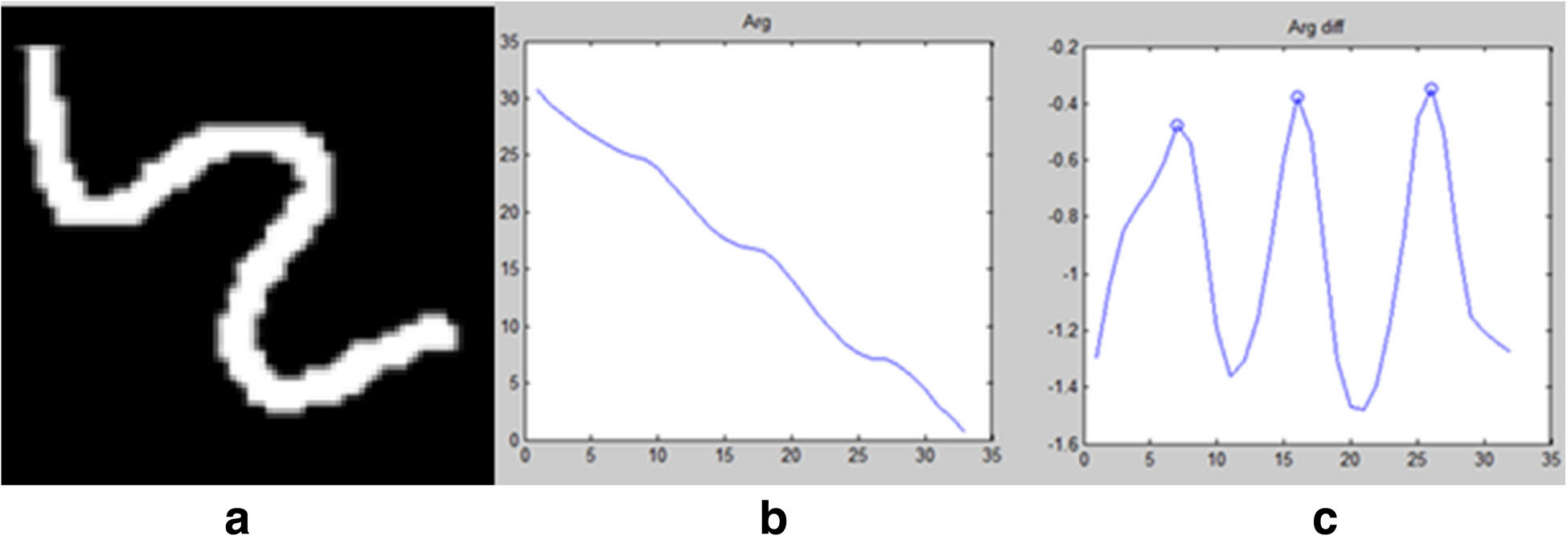
**a** A simulated vessel with three evident turning points. **b** The argument of its integrated voltage sequence. **c** The derivative argument of its integrated voltage sequence (turning points marked in circle)

Our proposed circuit conversion model is a feasible and effective way to transform the vascular characteristics (thickness and curvature) to integrated voltage sequences, which is of physical meaning. For a vascular structure with large radius and rich in bifurcations, its complex impedance is high, so its weight in getting voltage distribution is usually high. In addition, its NSI value of the excitation voltage is also large, leading to its even higher voltage. From the perspective of the absolute value of voltage, a branch with faster descending of the voltage gradient (the voltage difference between the forward and current node) reflects its fast trend towards thinner area, as illustrated in Fig. 6. Given the voltage argument, if there is an evident change in the argument of the branch voltage sequence, it shows a large turning point in the branch, as illustrated in Fig. 7.

The proposed method is not only reasonable, but also with merits. Since the voltage is based on the overall response from the whole circuit, the result of voltage will not be drastically changed by a certain error node. Besides, in the conversion model, the use of NSI value as the voltage value for the excitation voltage source further emphasizes the regional characteristics. The larger NSI value means greater local influence of the node, thus having larger weight for voltage response and reflecting special characteristics in a branch. In addition, the application of superposition of voltage power can largely reduce the calculation complexity.

### Node matching in the branch

Using integrated voltage sequence, nodes in each branch can then be matched. The range of integrated voltage sequence *V*_*b*_ (from the sensed image) of a branch is firstly linearly scaled according to the range of *V*_*a*_ (paired branch from the reference image) by *V*_*b*_ ' = *kV*_*b*_ + *b* where *k* and *b* are constants.

For each point in *V*_*b*_ ', search its reasonable matching position in the *V*_*a*_ sequence. The deviation of position is described with three parameters: the leading node in matching, the voltage difference and the voltage difference base node. For each node *b*_*i*_ (voltage of *V*_*bi*_ ') to be matched, search in the close region (20% of node numbers in a branch, adjustable) for node whose *V*_*aj*_ ≤ *V*_*bi*_ ' and *V*_*a*(*j* + 1)_ > *V*_*b*_ ' as its leading node in matching. Then calculate *ΔV*_1_ = (*V*_*bi*_ ' − *V*_*aj*_)/(*V*_*a*(*j* + 1)_ − *V*_*aj*_) and *ΔV*_2_ = (*V*_*a*(*j* + 1)_ − *V*_*bi*_^'^)/(*V*_*a*(*j* + 1)_ − *V*_*aj*_). If *ΔV*_1_ is smaller than *ΔV*_2_, select *j* as voltage difference base node and *ΔV*_1_ as voltage difference, otherwise, select *j* + 1 as voltage difference base node and *ΔV*_2_ as the voltage difference. The matching position (with three parameters) of all matching points *i* in sequence *b* can be acquired.

### Image fusion

The last step is to combine corresponding nodes and branches in a fused image. As the integrated voltage sequence represents the structural features of the branches at different locations, the voltage difference can be understood as the deviation of locations. The voltage difference, which is the ratio of the voltage deviated from the voltage between the leading node and the subsequent node (the forward node in the branch direction), represents the deviation in distance between the leading node and the succeeding node. The base node determines the direction of deviation.

Each of the corresponding node from sensed image is marked according to deviation in the reference graph network to form a fusion graph. In addition to marking the corresponding nodes, those unmatched branches also need to be marked in fusion image to present their unique information. These branches have one end node that has correspondence node already registered, using that node as reference, shifting the unique branches to these registered positions to fuse these branches.

### Computational setup

We ran all the algorithms on a 2.9 GHz dual-core 64-bit machine with 16 GB RAM. All 2D image registrations were implemented in a combination of Matlab and Mex-C++ functions. In 3D image registrations, we used VTK for easier visualization and C++ for algorithm implementation.

## Results

To test effectiveness and efficiency of our method, we use one set of simulated 2D images, one set of 2D retinal fundus images and one set of 3D Time-of-Flight Magnetic resonance angiography images as test data. For a better comparison between other state-of-the art vascular image registration methods, we tested along with a graph-based approach named Active Testing Search for Robust Graph Matching (ATS-RGM) [18] with both fine and coarse alignment and a feature-based approach named Coherent Point Drift (CPD) [15].

In our method, we use the default parameter mentioned previously. In ATS-RGM algorithm, we set *scale_factor =* 20, other parameters using the case of ‘retina’ in the paper. For CPD algorithm, we use a non-rigid configuration of the algorithm for all the experiments. We set *λ* = 3, *β* = 3 and outliers = 0.2.

### Simulation image

**Fig. 8:**
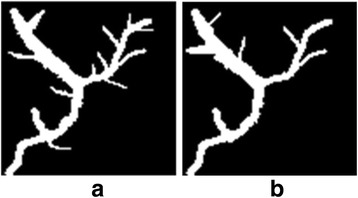
Example of a set of simulated images. **a** Reference image (**b**) Sensed image

As an example, Fig. 8 is a set of simulated 2D vascular images. There are several small interference branches near bifurcations of large structures in the reference image (Fig. 8a).

**Fig. 9:**
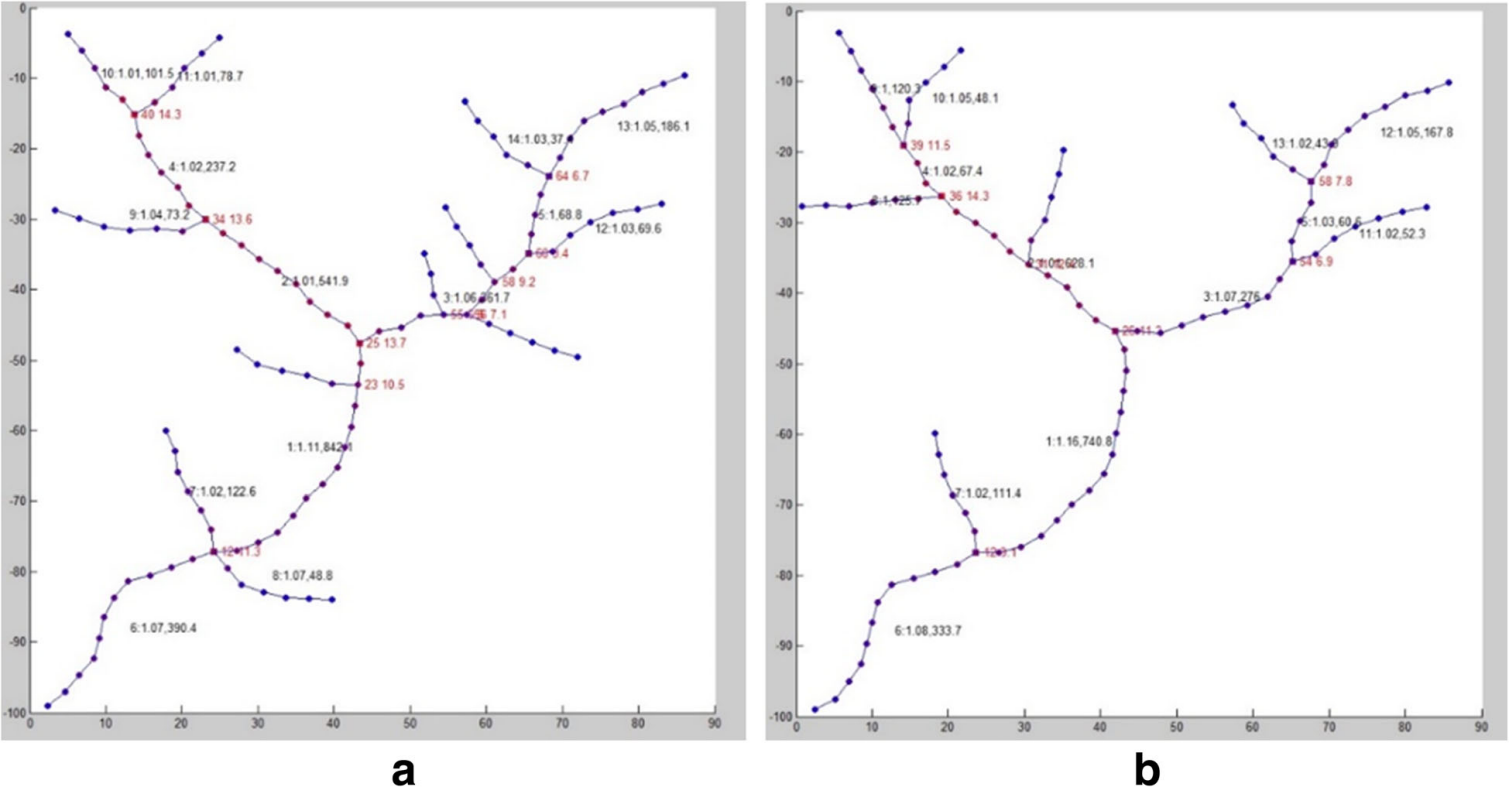
Matching result of structurally salient points (square nodes marked with id and NSI in *red*) and branches (marked with id, tortuosity and total radius of branch in *black*) in reference graph (**a**) and sensed graph (**b**)

After Open-curve snake tracing, we can get graph network with node number of 117 and 91 respectively. By calculating the NSI of nodes, the structurally salient points (square nodes) are matched, as shown in Fig. 9.

In Fig. 9, the square nodes are the structurally salient points (with id and NSI value marked in red) of the graph network after matching. The NSI of these square nodes are local maximum to avoid being mismatched by other interfering small branches in the near region. For example, the node id 25 in the reference graph is larger in NSI value than that of node 23 by 3.2, becoming the matching target.

According to the network decomposition method, the branches are sequentially divided and matched. Second kind of branches are matched according to sequential matching criteria.

Taking node id 12 from the reference graph as an example, its branching properties are shown in Table 1. It is found that the difference between the branch id 1 and 8 in the reference graph is less than 60° so that it needs further information to judge correspondence. And then sum radius of nodes in each branch is calculated, finding the difference is beyond allowable percentage of 30%, which is large enough to differentiate the correspondence.

**Table 1:** Branches starting from node id 12 in reference image and sensed image

Reference image				Sensed image			
Branch id	Tortuosity	sum radius of nodes	forward direction of starting point	Branch id	Tortuosity	sum radius of nodes	forward direction of starting point
1	1.115	842.062	0.864	1	1.150	875.634	3.383
7	1.017	122.590	95.907	7	1.019	108.525	90.977
6	1.074	390.403	200.983	6	1.087	365.629	205.201
8	1.072	48.812	307.179				

The node matching within the branch is performed for each set of branches. The matching position determined by three parameters (leading node, the voltage difference and the voltage difference base node) is shown in Table 2.

**Table 2:** Nodes matching of branch id 6 in reference image and sensed image

Branch id from sensed image	leading node in reference image	voltage difference	voltage difference base node in reference image
1	1	0.00%	1
2	2	21.04%	2
3	3	25.85%	3
4	4	8.64%	4
5	5	8.05%	4
6	6	15.00%	5
7	7	6.50%	6
8	8	3.56%	8
9	9	1.85%	8
10	10	7.62%	9
11	11	0.00%	11

**Fig. 10:**
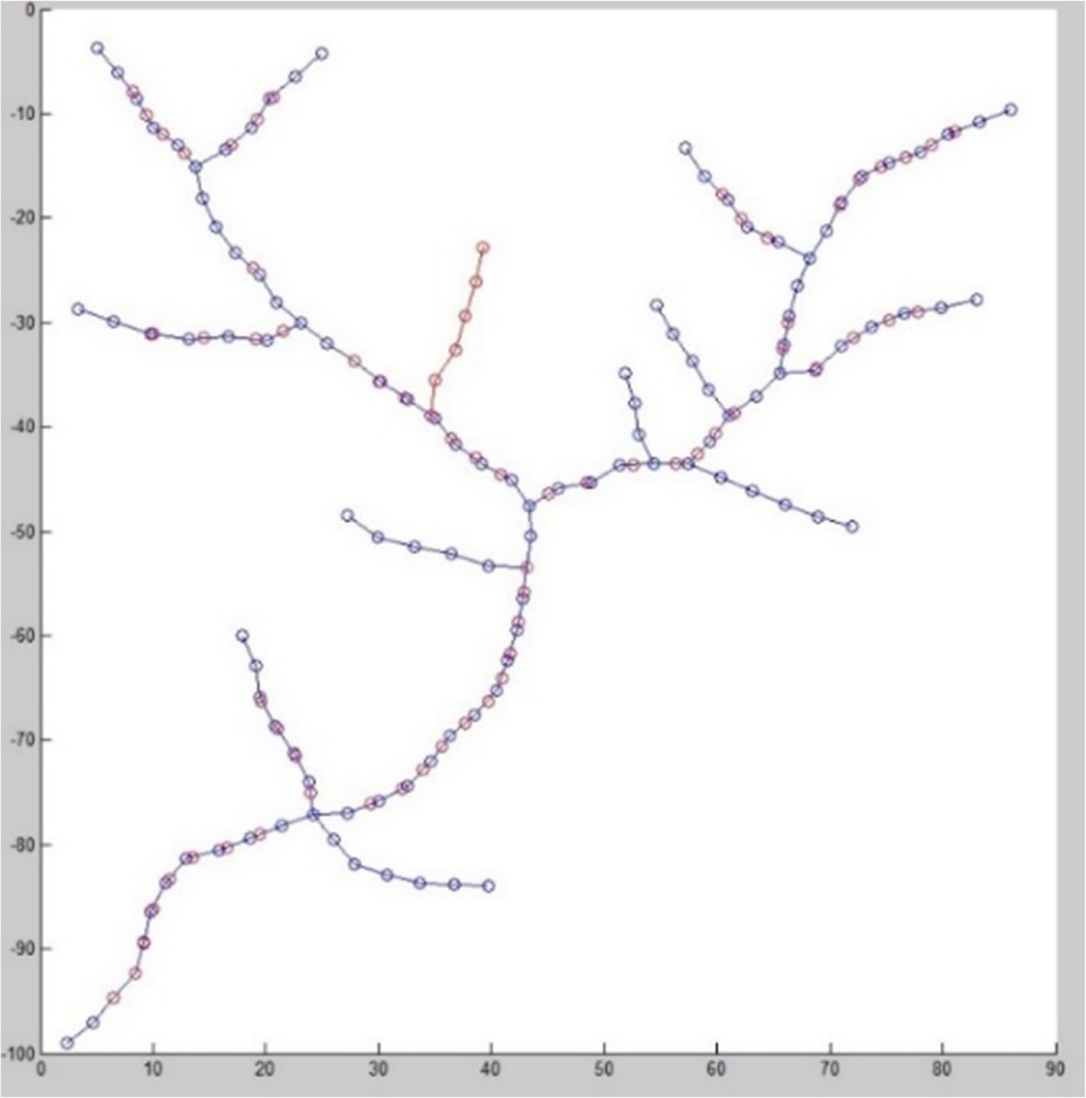
Fusion graph after registration (*blue nodes* for reference nodes, *red nodes* for fused graph)

The matching of each node is obtained by matching each branch in turn, and the positional deviation between two graphs is plotted on the fusion graph shown in Fig. 10.

There are unique unmatched branches in the sensed graph, which are added in the fusion graph. For example, the branch starting from node id 31 in sensed graph is shifted with same deviation of node 31 (4.1398 and 3.0534 in *x* and *y* direction) to be added in fusion graph.

After image registration, the same information in both images is strengthened, and the unique information from each image is fused.

### Clinical image

**Fig. 11:**
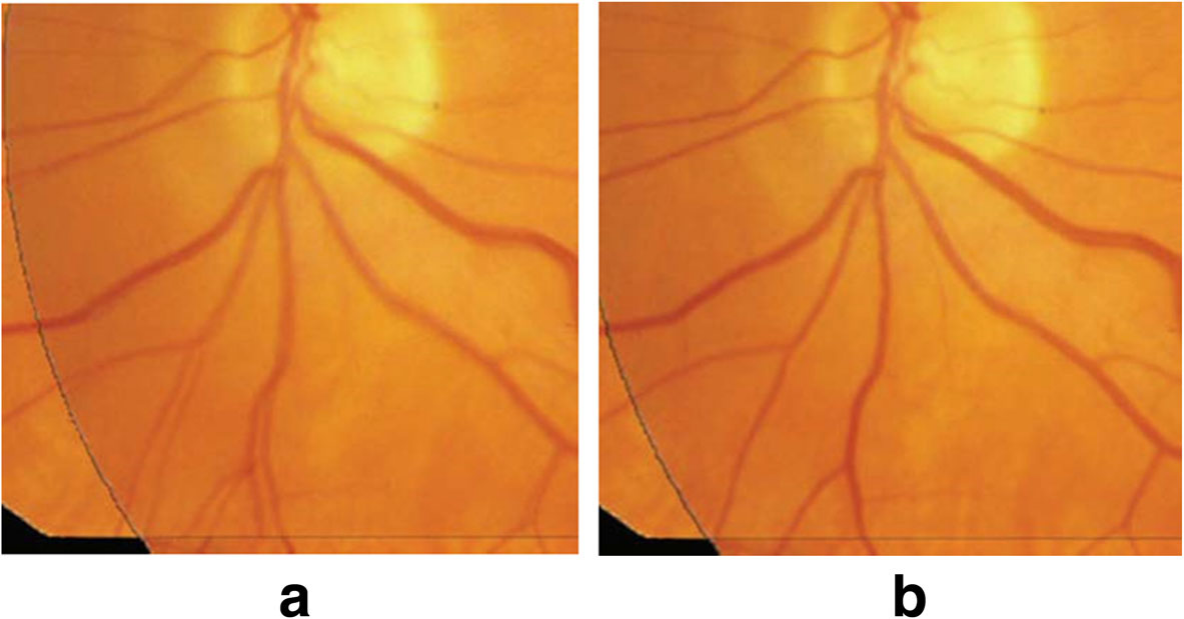
A set of retinal fundus images for registration. **a** Reference image (**b**) Sensed image

The registration of the clinical images is similar to the simulation images. A set of retinal fundus images is shown in Fig. 11 which is from Deng K et al.^1^ [17].

In this set of images, registration should be mainly focused on how to match a number of blood vessels from the same branching point. In our method, structurally salient points are matched based on regionally maximum NSI, which is stable in finding corresponding points. And branch matching is also accurate and effective by branch sequential matching criteria mentioned above in this case.

**Fig. 12:**
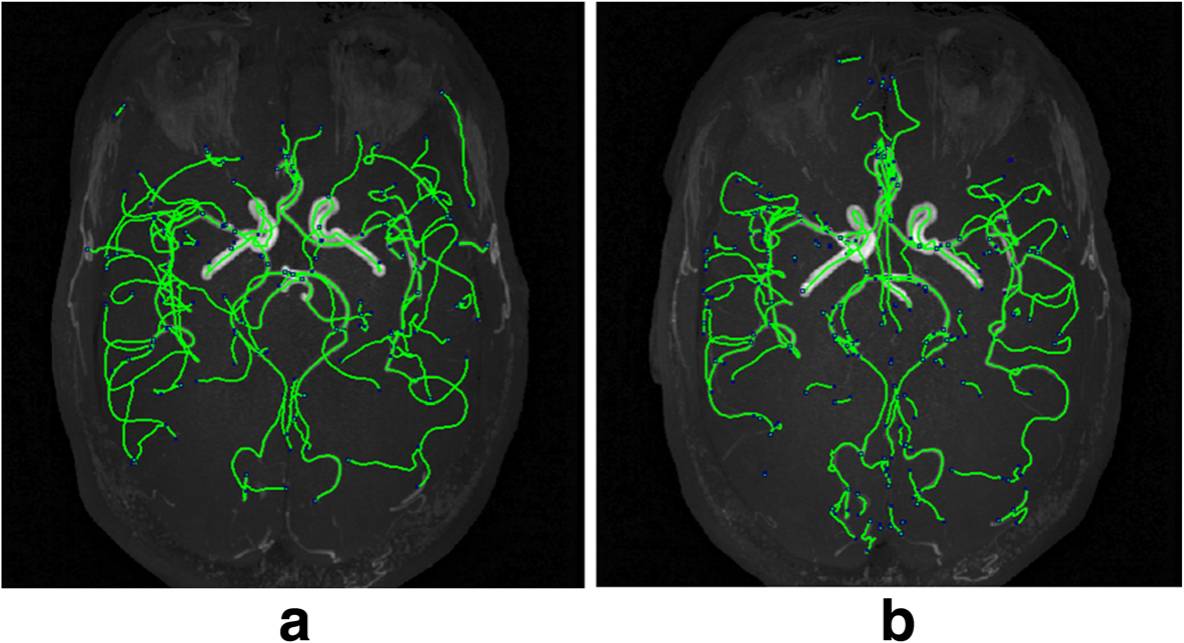
3D TOF-MRA images in MIP view with tracing result painted in *green lines*. **a** Reference image (**b**) Sensed image

Another set of Time-of-Flight Magnetic resonance angiography images(TOF-MRA) acquired from Vascular Imaging Lab in University of Washington is used to test our method in 3D images. The subject was taken twice scan for the same brain region within a week. Maximum Intensity projection (MIP) of both images are shown in Fig. 12. With open-snake tracing method, their traces are extracted and shown in green lines in both images.

As mentioned before, our registration method is based on graph network rather than directly using image intensity, it is almost with same complexity for 3D and 2D images. Note that the isolated structures in both images are not registered in our experiment as they are likely to be noises or trivial vessels.

**Fig. 13:**
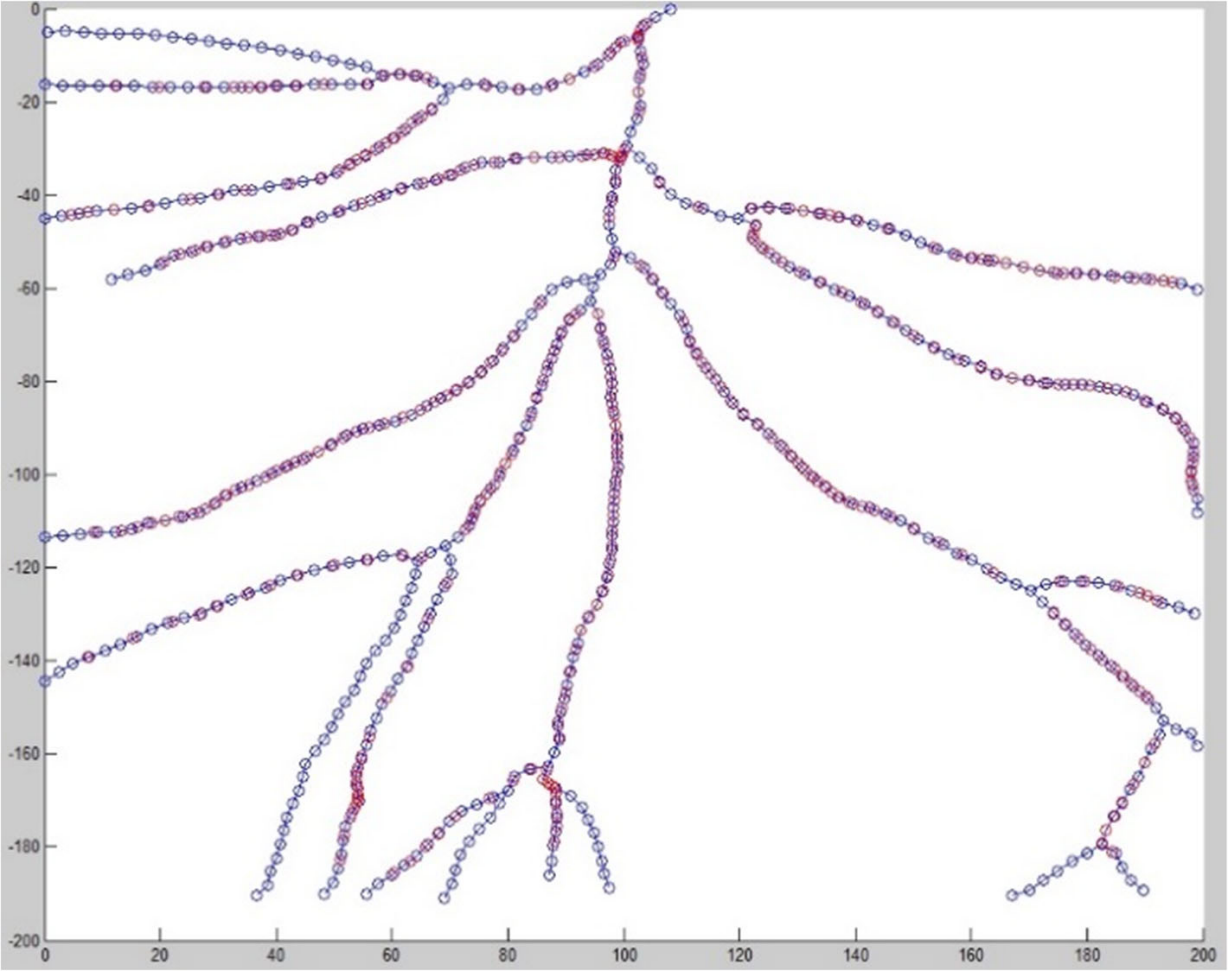
Fused graph after registration (blue nodes for reference nodes, red nodes for fused graph)

**Fig. 14:**
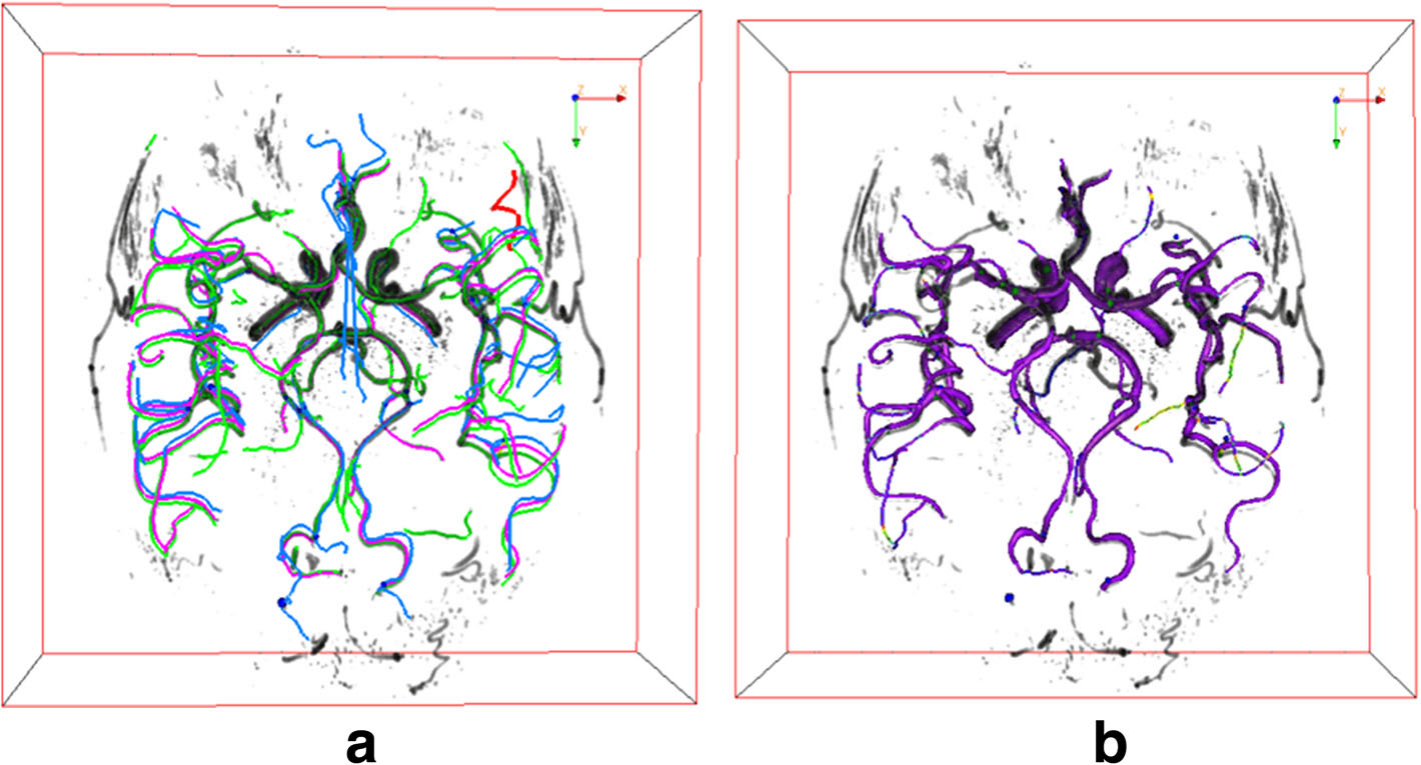
**a** registration result for 3D TOF-MRA images (*green lines* from reference image, *blue lines* from sensed image, purple line for fused image) (**b**) Fused vascular structures from the registration of TOF-MRA after surface rendering

Due to the similar process, we directly show the registration result in Fig. 13 and 14a. We can further use visualization tool to render the fused vessels shown in Fig. 14b.

### Calculation

We tested the speed of each algorithm in processing 2D image registrations. The fusion image along with processing time is listed in Table 3. Blue and red nodes from our method, blue and purple lines from ATS-RGM coarse alignment, blue and red lines from ATS-RGM fine alignment, blue and yellow lines from CPD are reference and fused nodes or lines respectively. Green circles from ATS-RGM coarse alignment are control points. * means the automatic registration for that algorithm is failed to find corresponding control points, so manual correction is needed. As mentioned in [18], there are two steps of alignment in ATS-RGM, and the processing time for fine alignment based on coarse alignment does not include the time for coarse alignment. It takes less than one second for our method to register both of the image sets, while CPD method needs several seconds and ATS-RGM is much more time-consuming.

**Table 3:**
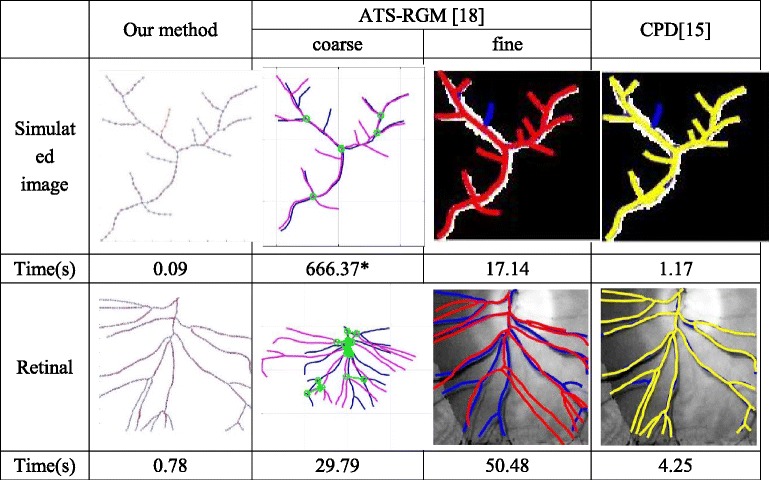
Fusion image and processing time of 2D images by our method, ATS-RGM and CPD

### Fault tolerance

We tried to change the radius of a certain node in the branch, and tested the influence of the variation on the integrated voltage sequence of the branch. Taking the branch 1 on Fig. 9a as an example, the radius of node id 10 was changed from 2.2037 to 0.01. This kind of error is possibly occurred during errors in imaging. The influence of the integrated voltage sequence is shown in Table 4. We find the difference of voltage before and after the error occurs of all nodes is less than 1%. Moreover, it is found that the region with larger difference is only at the end of the branch, and it does not cause obvious interference to the first half of branch which has more obvious branching features.

**Table 4:** Difference in integrated voltage sequence in percentage after error occurs

Node id	1	2	3	4	5	6	7	8	9	10
Voltage difference	0.0000%	0.0000%	-0.0005%	-0.0008%	-0.0011%	-0.0016%	-0.0021%	-0.0024%	-0.0031%	-0.0035%
Node id	11	12	13	14	15	16	17	18	19	20
Voltage difference	-0.0041%	-0.0046%	-0.0051%	-0.0060%	-0.0066%	-0.0073%	-0.0081%	-0.0092%	-0.0109%	-0.0119%
Node id	21	22	23	24	25	26	27	28	29	30
Voltage difference	-0.0129%	-0.0142%	-0.0159%	-0.0183%	-0.0207%	0.1884%	0.1983%	0.2132%	0.2328%	0.2573%
Node id	31	32	33	34						
Voltage difference	0.2994%	0.3599%	0.4452%	0.6082%						

## Discussion

Our method is an improved graph-based registration method, which not only has the benefits of other graph-based methods, but also has more advantages in stability, fault tolerance and low computation complexity.

### Stability

In our method, we use Network Structure Index to identify corresponding structural salient points, which is effective in determining interfere branches near bifurcation area. For interfere branches are comparably low in NSI due to its low weight and small connections, the main branches are not matched mistakenly in all three cases shown in last section, such as branches connected with node id 25 in Fig. 9a.

Compared with using geodesic distance in determining control points in ATS-RGM coarse alignment, when there are interference branches near bifurcation, such as the situations shown in 2D registration cases above, the ATS-RGM is easy to make wrong correspondence, leading to the failure in simulated image set and large deviation in the retinal set.

CPD method deals poorly when there are missing parts, such as the failure of matching one of the bottom branches in the top right image of Table 3, which is a common problem for feature-based approaches.

### Fault tolerance

We utilize the voltage conversion model, which has the advantage of high fault tolerance based on the structure of circuit. Because of the chain structure of voltage sources in circuit, if there is an evident error in one source, adjacent voltage sources can still describe its local characteristics without losing much local information. In addition, the voltage source excitation value is the NSI value of this node, which reflects the characteristics of the node within its neighborhood, and NSI value will not change drastically when a certain node in the region is changed, further ensuring the accurate description to a branch when there is a certain error node. Results in Table 4 have shown the impact of the integrated voltage sequence when a node is drastically changed is less than 1%.

### Calculation

Our method registers in graph-based approach, which avoids the direct use of pixel value. The example 2D image size is 100 × 100, containing 10,000 pixels. After the conversion to graph network, we only need to calculate based on more than 100 points and more than 100 connections. In addition, our method decomposes the network into several branches by the stable identification and matching of the structurally salient points based on network structure, further decreasing number of points to only a few dozens in each branch. Network decomposition is also beneficial in processing large scale graphs.

Except for the step of calculating gradient vector flow and vesselness which are used in Open-curve snake tracing, the NSI calculation and circuit conversion model are all based on simple summation and multiplication, which is fast to implement in program.

From the results in Table 3, our method is at least hundred times faster than ATS-RGM and five times faster than CPD.

However, when there is large initial rotation, such that the corresponding structural salient points are beyond the allowable distance we defined, our method will be ineffective. Similar situation will also appear in CPD as stated in their paper.

## Conclusion

In order to overcome the shortcomings of high noise and large differences in registration of vascular images, as well as to achieve efficiency in registration, a novel and effective method based on network structure and circuit simulation is proposed in this paper.

In our method, both reference and sensed images are converted to graph networks, which are decomposed by Network Structure Index. Branches segmented from network are then matched by sequential matching criteria. A circuit conversion modal is used to further convert graph network to circuit, the voltage response of which is used for node matching and image fusion.

By implementing the method on both simulated and clinical image sets, we find this registration method can effectively establish the correspondence between nodes and generate a fused graph network, with calculation time significantly lower than two state-of-the-art algorithms.

We mainly focused on vascular registration of retinal and MRA images in this paper. However, for other tubular structures or multi-modality image registration, this registration method still has much value and can be further researched in further work. In addition, our method is based on the graph network generated from Open-curve snake tracing method, the application scope of which has not been fully investigated.

In summary, the proposed vascular image registration method based on network structure and circuit simulation is a useful complement to the current mainstream image registration methods.

## Abbreviations

ATS-RGM: Active testing search for robust graph matching
CPD: Coherent point drift
NSI: Network structure index

## Footnotes

1 Copyright © 2010 Kexin Deng et al. This is an open access article distributed under the Creative Commons Attribution License, which permits unrestricted use, distribution, and reproduction in any medium, provided the original work is properly cited.
